# Transitioning to midwifery models of care: Implementation insights from focus groups with healthcare leaders in two African countries

**DOI:** 10.4102/phcfm.v18i1.5238

**Published:** 2026-02-19

**Authors:** Lise-Lotte Franklin Larsson, Solomon Hailemeskel, Helena Lindgren, Ulrika Byrskog, Michael B. Wells, Johanna Blomgren, Joyce Jebet Cheptum, Kaddy Ceesay, Kerstin Erlandsson

**Affiliations:** 1 Department of Sexual Reproductive Health, School of Health and Welfare, Dalarna University, Falun, Sweden; 2 Department of Midwifery, School of Nursing and Midwifery, Asrat Woldeyes Health Science Campus, Debre Berhan University, Debre Berhan, Ethiopia; 3 Department of Health Promotion, Sophiahemmet University, Stockholm, Sweden; 4 Department of Global Public Health, Karolinska Institute, Stockholm, Sweden; 5 School of Nursing and Midwifery, Aga Khan University, Aga Khan, Kenya

**Keywords:** midwifery, midwifery services, health services, accessibility, healthcare disparities

## Abstract

**Background:**

Midwifery models of care, endorsed by the World Health Organization (WHO), offer rights-based, person-centred care with proven benefits for safer maternal and newborn care worldwide. Despite these demonstrated benefits, the successful adoption of midwifery models of care depends on context-specific factors, making it essential to understand how healthcare leaders perceive and approach such transitions.

**Aim:**

To examine healthcare leaders’ perceptions of transitioning to midwifery models of care in Ethiopia and The Gambia, and to identify key barriers and facilitators influencing implementation.

**Setting:**

The study was conducted in Ethiopia and The Gambia within their respective maternal health system contexts, focusing on national and sub-national leadership perspectives.

**Methods:**

A qualitative study using open-ended, semi-structured interviews. An inductive thematic analysis was applied to explore leaders’ insights on midwifery models of care and system readiness for transition.

**Results:**

Facilitators included midwives’ active advocacy for women’s rights and strong community engagement in maternal health decision-making. Common barriers across both countries were workforce shortages, limited health system infrastructure, donor dependency, and persistent gaps in access for rural populations. Additional barriers included inadequate transport networks, geographic inaccessibility, financial challenges limiting women’s ability to reach skilled care, hierarchical governance structures that restrict midwives’ professional autonomy, insufficient facility readiness, and cultural resistance to evidence-based midwifery practices.

**Conclusion:**

Transitioning to midwifery-led models of care requires context-specific strategies aligned with the World Health Organization implementation guidance, with particular attention to strengthening infrastructure, financing, and workforce capacity.

**Contribution:**

The study underscores the need to integrate midwives more fully into policy and governance structures. Strengthening their leadership and advocacy roles may enhance the visibility, influence, and overall contribution of the midwifery profession within national health systems.

## Introduction

Despite considerable progress over recent decades, preventable maternal and newborn deaths remain unacceptably high worldwide, with disproportionate burdens in low- and middle-income countries.^1,2^ In addition to mortality, millions of women and newborns experience lifelong severe morbidities, including eclampsia, and cardiovascular complications, while stillbirth rates remain largely stagnant.^1,3^ These risks are especially pronounced among adolescents, women with limited access to sexual and reproductive health and rights services and those living in humanitarian or fragile settings.^4,5,6^ A substantial proportion of maternal and newborn deaths, including stillbirths, are preventable with timely access to high-quality, respectful and evidence-based care. While expanding service coverage remains essential, growing evidence shows that poor-quality care contributes to more deaths than limited access in many settings.^7^

In low- and middle-income countries, over half of maternal deaths and more than 60% of neonatal deaths could be prevented through access to high-quality midwifery services.^7^ Poor-quality care, including delayed interventions, misdiagnoses and inadequate clinical management, can directly contribute to fatal outcomes for mothers and newborns.^8,9^ However, women and newborns frequently encounter mistreatment, disrespect and overmedicalisation during care. While women may not directly die from experiences of disrespect or mistreatment, poor quality of care has been shown to contribute to preventable maternal morbidity and mortality.^10^ Overuse of interventions, such as non-indicated caesarean sections, routine episiotomy and unnecessary augmentation, has been associated with adverse outcomes and escalating health system costs. Such practices undermine trust in the health system and hinder progress towards universal health coverage.^11^

Midwifery models of care offer a cost-effective, evidence-based pathway to improve outcomes for women and newborns while reducing unnecessary interventions.^12^ Designing a model of care requires defining core service delivery elements, ensuring continuity across the maternal and newborn health continuum, pre-pregnancy, antenatal, intrapartum and postnatal care.^13^ A midwifery model of care places professional midwives as the primary care providers, working autonomously within their scope of practice while collaborating effectively with other professionals through robust referral systems.^14^ Grounded in person-centred, respectful and relationship-based care, these models optimise health outcomes and women’s experiences while maintaining the lowest possible intervention rates consistent with safety.^14^

Systematic reviews confirm that midwife-led continuity models reduce unnecessary medical interventions, improve maternal satisfaction and are associated with equal or better safety outcomes compared to other care models.^12^ The World Health Organization (WHO) advocates for transitioning health systems towards midwifery models of care as part of strengthening primary health care and achieving universal health coverage.^15^ Transitioning to midwifery models of care entails shifting from fragmented, risk-oriented approaches to integrated, equitable and respectful services coordinated by midwives within interdisciplinary teams.^12,16,17^ This transition aligns with WHO’s primary health care principles, promoting comprehensive, integrated and context-adapted service delivery.^14^ In these models, midwives work collaboratively with obstetricians, paediatricians and other specialists, ensuring timely referral and continuity of care in the event of complications. Successful implementation requires alignment with national health priorities, investment in workforce development, supportive regulation and societal acceptance of midwifery as a profession. Despite a global consensus on the benefits of midwifery models of care,^14,15^ barriers to their implementation remain insufficiently implemented in many countries, limiting the ability of health systems to scale up effective interventions and improve maternal and newborn outcomes. Health system structures, professional hierarchies, limited policy frameworks, resource constraints and socio-cultural perceptions of midwifery can hinder transition efforts.^12,18^ These perspectives have not been explored from the viewpoint of leaders in Ethiopia and The Gambia, whose insights are vital as they help drive and influence healthcare systems and organisational change. Conversely, facilitators such as political commitment, supportive leadership, interprofessional collaboration and community engagement may accelerate progress.^19,20,21^ Understanding these context-specific barriers and facilitators further is critical for tailoring implementation strategies that are both effective and sustainable.^14,15^

Ethiopia and The Gambia both employ tiered healthcare systems, though their structures differ. Ethiopia’s system consists of four tiers: health posts, health centres, primary hospitals and referral hospitals.^22^ In contrast, The Gambia operates a three-tier system comprising primary care: health posts, minor health centres, secondary care: major health centres and district hospitals.^23^ Both countries integrate midwifery services into public healthcare, with midwives playing a central role in maternal and neonatal health. The structural differences between three-tiered and four-tiered healthcare systems have significant implications for midwifery services. In three-tier systems, such as The Gambia, midwives are primarily concentrated at secondary and tertiary levels, limiting access for rural populations and creating longer referral pathways.^23^ Conversely, four-tier systems, exemplified by Ethiopia, enable greater decentralisation, placing midwives closer to communities. However, equity-focused policies are especially critical in Ethiopia, given the large rural–urban and wealth disparities.^22^ Because of the variation in health system capacity, interventions need to be context specific: what works in Ethiopia may not directly translate to The Gambia. Midwives in both countries face systemic barriers that limit their ability to advocate for quality maternal care. Despite midwives’ frontline role, they are often excluded from decision-making, contributing to persistent gaps in service delivery.^8,11,17,22,23^ Challenges such as midwife shortages, under-resourced facilities, donor dependency, limited rural access and socio-cultural barriers to modern care are also identified.^2,4,13,22,23^ With this background, the aim of this study is to examine healthcare leaders’ perceptions on transitioning to midwifery models of care in Ethiopia and The Gambia, identifying implementation barriers and facilitators in these countries.

## Research methods and design

### Study design

A qualitative, inductive, thematic analysis was conducted using open-ended interviews on midwifery models of care.^12^ Data were analysed thematically with attention to both manifest and latent content following the approach described by Graneheim and Lundman.^24,25^

### Setting

Both countries^22,23^ face persistent shortages of skilled midwives; however, Ethiopia’s Health Extension Program (HEP) partially mitigates this through task-shifting and community-based care. This structure improves coverage but introduces challenges in maintaining quality, supervision and resource allocation across dispersed tiers.^22^ The Gambia struggles with infrastructure gaps and financial constraints.^23^ Subsequently, maternal care, including antenatal, intrapartum and postpartum care, is often delivered by multiple providers with different professions,^22,26^ which can lead to fragmented communication, limited record sharing, inconsistent advice and an increased risk of missed warning signs.^15^ These challenges have prompted interest in transitioning towards midwifery-led models of care.

### Participants

Twenty healthcare leaders with experience from clinical, academic and policy work were enrolled in an online Leadership Capacity Building Training Program in the academic year of 2023/2024 by the Karolinska Institute.^27^ The participants were fluent in English and had qualifications in midwifery, nursing, public health and medicine. All participants were informed that participation in the focus group discussions (FGD) was voluntary, the FGD’s would be recorded, and the recordings would be used for research purposes. All participants were encouraged to sign the written consent form, which they were asked to return to the facilitators of the FGD.

### Data collection

Three FGDs were conducted online with a total of 20 participants, 6–7 participants in each FGD. Consisting of a combination of leaders with expertise in clinical practice, education and policy management. The discussions lasted between one and one and a half hours.

The FGDs were facilitated by three PhD holders who also led the Capacity Building Training Program. In line with the FGD method,^25^ they were supported by research assistants to ensure that all participants’ voices were heard and that trustworthy results were obtained.

A topic guide was developed with open-ended questions allowing the participants to express and elaborate on their perceptions of transitioning to midwifery models of care (see Box 1).^24,25^ The questions were organised around key thematic areas: perceptions of resistance to evidence-based practices, perceived barriers and facilitators to adoption and scale-up, the role of patients, communities and managers, as well as the influence of work environment and work structure. Participants were also invited to share additional reflections on context-specific factors affecting implementation. This structure allowed for rich, comparative insights across the two settings while maintaining flexibility for emerging themes. All FGDs were audio-recorded with participants’ consent and subsequently transcribed using Otter.ai by three of the authors who were not involved in data collection.

BOX 1 Topic guide for focus group discussions.

Purpose of the focus group discussions

The purpose of this Focus Group Discussion is to examine healthcare leaders’ perspectives on transitioning to midwifery models of care in Ethiopia and The Gambia to identify barriers and facilitators.

**Country:** __________________________

**Topic Areas and Questions**

1. **Perceptions of resistance to evidence-based practices**
  - In your view, what are the reasons why midwives, doctors, and nurses may be reluctant to adopt evidence-based practices in maternal and child health?
    ▪ At your workplace?
    ▪ In general, in your country?
2. **Perceived barriers**
  - What barriers do you think influence the scaling up of midwifery models of care in maternal and child health in your country?
3. **Facilitators to adoption and scale-up**
  - In your opinion, what could motivate midwives, doctors, and nurses in your country to support and participate in scaling up midwifery models of care?
4. **Barriers to adoption and scale-up**
  - What factors might discourage or prevent healthcare providers from scaling up midwifery models of care?
5. **Role of patients, communities, and managers**
  - Do midwives, nurses, and doctors feel encouraged by patients, communities, or supported by managers (either in community health settings or hospitals) to provide midwifery models of care?
  - Did these forms of encouragement – or lack thereof – affect their decision to work in an evidence-based way? How?
  - Do you think these aspects influence the scaling up of midwifery models of care? How?
6. **Work environment**
  - How would you describe your working environment?
  - Has this environment in any way influenced the scaling up of midwifery models of care?
7. **Work structure**
  - Has the way work is organised at your workplace influenced the scaling up of midwifery models of care? How?
8. **Additional reflections**
  - Is there anything else you would like to share about barriers or facilitators that influence the scaling up of midwifery models of care?

### Data analysis

A thematic analysis (Table 1), focusing on the manifest and latent content of the data, was conducted. Transcripts were analysed using the steps outlined by Graneheim and Lundman.^24^ Initially, the texts were read in their entirety to gain an overall understanding. Then, meaning units were identified, condensed and coded. These codes were organised into groups, and similar groups were combined into subcategories that reflect patterns emerging in the data. The sub-themes were further abstracted into an overarching theme. The transcripts were independently read and analysed, and the process of thematisation was iteratively refined through collaborative discussions among the research team to enhance analytical rigour and reduce potential bias.^25^

**TABLE 1:** Examples of the analysis process.

Meaning unit	Condensed meaning unit	Code	Sub-theme	Theme
Geographical and infrastructural constraints and poor road conditions, long distances to health facilities, especially in rural areas.	Inadequate resources for implementing best practices	Inadequate infrastructure	Navigating structural, financial, and cultural realities in midwifery care transition	Structural and systemic barriers must be addressed to enable equitable, rights-based midwifery models of care
Creating awareness about this continuum of care is the first step. Once understood, the community can accept it because it benefits women and is proven effective.	Enhancing awareness of the care continuum constitutes an essential initial step. Once this concept is understood, communities are more likely to endorse it as an evidence-based, person-centred approach that optimises outcomes for women	Enhancing and implementing person-centred care	Enacting high-quality, evidence-based, and person-centred midwifery philosophy	Structural and systemic barriers must be addressed to enable equitable, rights-based midwifery models of care
If policymakers believe and accept this model as a standard and include it in the healthcare system, most challenges can be managed.	If policymakers endorse this model as a standard and integrate it into the healthcare system, many existing challenges can be effectively mitigated	Policy influence can enhance midwives’ autonomy	Strengthening midwifery autonomy and integration within collaborative health systems	Structural and systemic barriers must be addressed to enable equitable, rights-based midwifery models of care

### Ethical considerations

Ethical clearance to conduct this study was obtained from the University of Dalana, Department of Sexual Reproductive Health, School of Health and Welfare. Ethical approval was obtained from Debre Berhan University, Ethiopia (Ref. No: IRB 01/13/2017), The Gambia government/MRC joint ethics committee (project ID7 ref: 28333) and Swedish Ethical Review Authority (Dnr 2025-02187-01). All participants’ identities were kept confidential throughout the process. The participants were informed that it was voluntary to participate in the focus group discussion (FGD) and that they were free to leave at any moment without providing any reason. All participants were fully informed about the study’s purpose, procedures and potential risks and voluntariness before participating. In this case, the participants consented to participate in the study by taking part in the online FGD.

## Results

The overarching main theme was *Addressing Structural and Systemic Barriers to Achieve Equitable, Rights-Based Midwifery Care Models*. It emerged through three sub-themes: *Navigating Structural, Financial and Cultural Realities in Midwifery Care Transition, Enacting High-Quality, Evidence-Based and Person-Centred Midwifery Philosophy* and *Strengthening Midwifery Autonomy and Integration Within Collaborative Health Systems*.

### Addressing structural and systemic barriers to achieve equitable, rights-based midwifery care models

Facilitators for implementing midwifery care models included recognising midwives as key agents of gender equality and reproductive rights, providing community-based and culturally responsive services and promoting interdisciplinary collaboration. Team-based approaches with respect for different professions, clear roles and shared protocols were seen as essential for equitable, continuous, person-centred care. Conversely, barriers were mainly structural and systemic, such as geographic inaccessibility, financial hardship and organisational hierarchies limiting midwives’ autonomy and access to resources.

#### Navigating structural, financial and cultural realities in midwifery care transition

Significant barriers to maternal healthcare were rooted in structural and geographic constraints. Public services were often inaccessible and private care unaffordable, leaving some women to give birth at home. Distance was a critical factor: although guidelines recommend living within five kilometres of a facility, many women live far beyond this range, sometimes delivering en route. This gap between policy and reality delays care and increases maternal risk. Normative guidelines fail to account for geographic isolation and socioeconomic hardship, undermining policy effectiveness and heightening vulnerability. In one FGD, this was described as:

‘Public services inaccessible and private services unaffordable … some women … give birth at home due to lack of options “… for pregnant mothers to access care, it has to do with distance”. The guideline says everyone should stay at least 5 kilometres away from a health care centre, but that’s not the reality on the ground. Still, some are very far from the facilities, and as a result, by the time they start off from their homes to the facility, some of them might deliver along the way because the facility is really far’. (FGD 3, female leadership, The Gambia)

The gap between policy directives and pregnant women’s realities leads to delayed care and, at times, childbirth en route to facilities. Guidelines overlook contextual challenges like geographic isolation and poverty, creating a misalignment that weakens policy effectiveness and heightens maternal vulnerability, increasing adverse birth outcomes.

Geographical distance, poor transport networks and seasonal inaccessibility were cited as recurring obstacles, particularly for rural populations. Even when health facilities exist, their accessibility is compromised by environmental and infrastructural limitations. Geographical and infrastructure limitations are further discussed in another FGD:

‘… geographical and infrastructural constraints and poor road conditions, [*including*] long distances to health facilities, especially in rural areas’. (FGD 2, female leader, Ethiopia)

The quote reflects a deeper issue of inequity in healthcare access, where rural women face systemic disadvantages because of infrastructural neglect. Such barriers affect the entire continuum of care, from antenatal to postnatal services, heightening maternal and newborn risk.

Some healthcare leaders highlighted the role of midwives as rights-based advocates, bridging policy ideals with community realities through culturally sensitive engagement. Midwives were seen as being able to bridge the gap between policy ideals and the local community, as described by one FGD as:

‘Midwife’s professional role as a rights-based advocate … community-based and culturally appropriate midwifery care’. (FGD 2, female leaders, Ethiopia)

Advocacy ideals, alongside community outreach, were seen as critical facilitators for improving uptake of midwifery-led care. However, Midwives’ decision-making power was described as curtailed by top–down directives from senior clinicians and administrators, leaving them unable to fully exercise their competencies even in routine care. Although midwives were seen as enablers of bridging policy ideals with the local community, reality was described differently in the following quote:

‘… they are not empowered enough. Even if they are being mistreated, they can’t speak. It is not up to the midwife to decide on things. At the facility, they are just there to obey; they cannot refuse or demand. In our culture, the power is with the husband, the father, or the community leaders. Even when labour starts, a woman cannot go to the hospital straight away without seeking consent. That really affects their access and acceptance of midwifery-led care’. (FGD3, female leaders, The Gambia)

The quote describes reality being opposite to the ideal of midwives having a rights-based advocate role in maternity care and highlights both midwives’ and women’s lack of empowerment in maternity care decisions. Explicitly, participants describe how women cannot speak up when mistreated and must seek consent from husbands, fathers or community leaders before accessing care. Midwives themselves have limited authority, expected only to obey institutional rules. This reflects entrenched patriarchal norms and hierarchical structures that restrict women’s autonomy and position midwives as passive actors. These dynamics reveal systemic power imbalances where cultural and institutional forces intersect to marginalise women, delay care and hinder acceptance of midwifery-led models, ultimately perpetuating inequity and vulnerability. Hierarchical decision-making structures in health facilities restrict midwives’ autonomy. Midwives cannot make independent decisions and lack basic equipment, which limits their ability to provide quality midwifery care. These structural limitations are described by participants as:

‘Hierarchical decision-making structure limits midwives’ autonomy. Even they have lack of basic equipment to enhance midwifery care. This is a structural limitation’. (FGD 1, female leaders, Ethiopia)

Such structural limitations meant that midwives not only had reduced scope to act independently but also lacked the necessary resources to deliver evidence-based care. As a result, their role was often relegated to following instructions rather than leading care, which the participants elaborated on as demotivating and as limiting the quality and timeliness of services for women.

Traditional beliefs surrounding childbirth are described as influencing women’s willingness to accept evidence-based practices. Specifically, some women refuse modern birthing positions because these practices conflict with cultural norms or evoke mistrust. This indicates that cultural perceptions and discomfort act as barriers to adopting recommended clinical interventions during labour and is expressed in one FGD as:

‘Traditional beliefs around childbirth influenced women’s acceptance of evidence-based practices, with some “refusing modern birthing positions … due to cultural discomfort or mistrust”’. (FGD 2, female leaders, Ethiopia)

The quote reflects a cultural resistance to evidence-based practices, where entrenched traditions and mistrust of modern health systems hinder the integration of evidence-based practices, potentially compromising maternal outcomes.

Although midwives often face limitations in practising their role as rights-based advocates who promote community-based and culturally appropriate care, the ideal of fulfilling this role remains strongly emphasised. This highlights that midwives are not only clinical practitioners but also advocates of equity and respect, working to ensure that maternal health services uphold human rights principles. One FGD expressed:

‘Midwife’s professional role as a rights-based advocate … community-based and culturally appropriate midwifery care’. (FGD 2, female leaders, Ethiopia)

Although midwives often face limitations in fully exercising their role, their perception of their professional responsibility extends beyond clinical tasks to include advocacy for equity and culturally respectful care. This reflects an aspiration for midwives to bridge systemic gaps and foster trust within communities. While their advocacy and community engagement are key facilitators of midwifery-led care, their limited empowerment and influence within midwifery healthcare structures and societal norms significantly hinder their ability to effect change.

#### Enacting high-quality, evidence-based and person-centred midwifery philosophy

Enacting high-quality, evidence-based and person-centred midwifery philosophy represents a holistic approach to maternal care that integrates clinical excellence with individualised support. This concept emphasises three interrelated dimensions: High-quality care – grounded in professional standards, safety and effectiveness, ensuring optimal maternal and neonatal care. Evidence-based practice – the application of current research and best practices to guide clinical decision-making, reducing unnecessary interventions and promoting physiological birth. Person-centred philosophy – recognising the woman as an active participant in her care, respecting her autonomy, cultural values and preferences throughout the childbirth continuum. This philosophy positions midwives as advocates for respectful, rights-based care, bridging evidence-based knowledge with cultural sensitivity. It challenges hierarchical and interventionist models by promoting shared decision-making and individualised care plans. Ultimately, enacting this philosophy requires systemic support, adequate resources and empowerment of midwives to practice autonomously within collaborative frameworks. Participants suggest that the successful implementation of a midwifery-led care model depends on policymakers’ acceptance and integration of this approach into the health system. They imply that systemic challenges, such as resource limitations, hierarchical structures and cultural barriers, could be mitigated if the model is recognised as a standard of care and supported at the policy level:

‘If policymakers believe and accept this model as a standard and include it in the healthcare system, most challenges can be managed’. (FGD 1, female leaders, Ethiopia)

The quote reflects a structural transformation through policy commitment, where institutional recognition and resource allocation are seen as prerequisites for overcoming barriers and achieving equitable, evidence-based maternal care.

Participants noted that women rarely request evidence-based practices such as dynamic birthing positions or immediate skin-to-skin contact, reflecting limited awareness, empowerment and cultural acceptance. This gap between recommended care and women’s expectations stems from cultural norms, inadequate health education and systemic barriers. For example, skin-to-skin care is resisted because of fears that the newborn will become cold. One FGD explicitly underscores the need for strategies that promote informed choice and person-centred midwifery care:

‘Women do not ask for dynamic birthing positions or skin-to-skin care’. (FGD 3, female leaders, The Gambia)

The quote highlights women’s limited agency and knowledge regarding evidence-based practices such as dynamic birthing positions and skin-to-skin contact. This lack of demand reflects cultural norms, inadequate health education and systemic barriers. Underlying these behaviours is a misalignment between recommended care and women’s expectations, which underscores the need for strategies that promote informed choice and culturally sensitive, person-centred midwifery care. For instance, resistance to skin-to-skin contact stems from cultural fears that the newborn will become cold. Further reflections on the consequences of this resistance, noting that it can delay early bonding and breastfeeding initiation, which may negatively impact neonatal health outcomes is expressed by participants as:

‘… that such resistance could delay early bonding and breastfeeding initiation, with potential downstream effects on neonatal health’. (FGD 3, female leaders, The Gambia)

The quote illustrates how cultural beliefs override evidence-based practices, limiting optimal maternal and neonatal outcomes. Participants stressed that raising awareness about the benefits of the care continuum is key to community acceptance. Education and communication are essential to bridge the gap between recommended practices and cultural norms and is discussed in one FGD as:

‘Creating awareness about this continuum of care is the first step. Once understood, the community can accept it because it benefits women and is proven effective’. (FGD 1, female leaders, Ethiopia)

The participants suggest that informed communities are more likely to embrace evidence-based, person-centred care models. This underscores the role of health education and participatory communication strategies in overcoming resistance and promoting equitable maternal care.

#### Strengthening midwifery autonomy and integration within collaborative health systems

Midwives’ autonomy means exercising independent clinical judgement within their scope of practice. Strengthening autonomy requires reducing hierarchical constraints and enabling midwives to lead care for low-risk pregnancies, advocate for women’s rights and apply evidence-based practices without interference. Integration into multidisciplinary teams promotes shared decision-making, clear roles and mutual respect, improving continuity of care. However, conflicts among nurses, midwives and doctors during decision-making remain a barrier to effective maternal care and are described as follows:

‘Conflicts between nurses, midwives and doctors in decision-making create barriers’ (FGD 2, female leaders, Ethiopia).

Fragmented teamwork and hierarchical dynamics are highlighted here and are likely to arise from hierarchical structures, unclear roles and competing professional perspectives. Such a work environment can delay interventions and compromise collaborative care and the implementation of midwifery-led care. To address these challenges, participants advocated systemic reforms that decentralise authority and formally recognise midwives as lead providers within their scope, free from structural constraints that limit midwives’ ability to exercise autonomous clinical judgement. The participants emphasise that policy-level endorsement is critical for the successful implementation of the midwifery-led care model. They suggest that if policymakers recognise and institutionalise this model as a standard within the health system, many existing challenges, such as resource shortages, hierarchical barriers and cultural resistance, could be effectively addressed:

‘If policymakers believe and accept this model as a standard and include it in the healthcare system, most challenges can be managed’. (FGD 1, female leaders, Ethiopia)

The participants’ discussion, illustrated in this quote, reflects a structural transformation where institutional recognition and resource allocation are seen as prerequisites for overcoming systemic barriers and achieving equitable, evidence-based maternal care.

## Discussion

This study reveals that despite global consensus on the benefits of midwifery-led, evidence-based care, implementation remains hindered by structural deficiencies, socio-cultural norms and knowledge gaps. Persistent geographic and resource limitations, coupled with restrictive gender dynamics and low health literacy, undermine equitable access and acceptance of recommended practices. These findings highlight the inadequacy of current health system strategies and underscore the need for systemic and cultural transformation to make midwifery-led care actionable rather than aspirational.

### Structural and geographical barriers

Persistent structural and geographical barriers significantly undermine the implementation of midwifery-led models of care in Ethiopia and The Gambia. Poor road infrastructure, long distances to health facilities and seasonal inaccessibility delay or prevent timely antenatal, intrapartum and postnatal care, contributing to elevated maternal mortality ratios, 289 and 267 deaths per 100 000 live births, respectively.^28^ These findings echo global evidence that geographic inequities remain a critical determinant of maternal health outcomes despite policy commitments to universal access.^13,14,15^ The WHO Implementation Guide for Midwifery Models of Care emphasises systematic documentation of such inequities to inform strategic responses.^15^ Elevating rural transport and facility access to national priorities and operationalising interventions such as maternity waiting homes, rural facility upgrades and transport subsidies could mitigate these barriers. Monitoring indicators, including travel times and seasonal service interruptions, is essential for accountability and progress tracking.

Financial hardship compounds these structural challenges, particularly where public services are limited and private care costs are prohibitive.^29^ This aligns with WHO’s guidance on sustainable financing,^15^ which calls for integrating cost analyses into midwifery plans and securing funding through national budgets, donors and innovative public–private partnerships. Policy actions such as expanding fee exemptions, piloting community-based insurance and incorporating transport vouchers could increase affordability and service uptake. Without such measures, structural inequities will continue to impede the scale-up of midwifery-led care.

### Socio-cultural norms and traditional beliefs

Socio-cultural norms and traditional beliefs emerged as powerful determinants of maternal health-seeking behaviour and acceptance of evidence-based practices.^30,31^ Women’s autonomy is frequently constrained by hierarchical decision-making within households, requiring consent from husbands or community leaders before accessing care.^31^ Cultural resistance to interventions such as skin-to-skin contact and dynamic birthing positions reflects deep-rooted traditions that conflict with biomedical recommendations.^30,31^ These findings underscore the inadequacy of purely clinical strategies and highlight the need for culturally adaptive approaches. The World Health Organization guidelines advocate for embedding respectful maternity care and cultural responsiveness within midwifery reforms.^15^ Co-development of educational materials that integrate biomedical and traditional perspectives, midwife training in cultural counselling and dialogue platforms that foster trust between communities and health providers are critical strategies.^32^ Monitoring indicators such as community trust scores and uptake of respectful care practices can provide meaningful measures of progress. Without addressing these socio-cultural dynamics, efforts to scale midwifery-led care risk perpetuating inequities and reinforcing gendered power imbalances.

### Knowledge and awareness gaps

Creating awareness about the continuum of care can be seen as the first step towards acceptance of midwifery-led care. Knowledge gaps among women regarding evidence-based practices – such as dynamic birthing positions and immediate skin-to-skin care – limit demand for interventions that improve maternal and newborn outcomes.^30,31^ This demand-side barrier reflects systemic neglect of health literacy within maternal health strategies. The World Health Organization emphasises community engagement and education as core components of midwifery implementation,^15^ yet these remain underdeveloped in many contexts. Targeted health literacy campaigns, peer education models and adolescent-focused maternal health education can bridge these gaps. Leveraging community health workers and midwives as advocates for evidence-based care ensures that information is culturally relevant and accessible.^30,31^ Monitoring uptake of recommended practices and shifts in health-seeking behaviours can inform iterative improvements. Addressing knowledge gaps is not merely an educational challenge but a prerequisite for empowering women to exercise agency in maternal care decisions.

### Cross-cutting systemic barriers and facilitators

Institutional and governance barriers, including hierarchical decision-making, limited facility readiness and inadequate equipment, restrict midwives’ autonomy and capacity to deliver respectful, evidence-based care.^19^ These findings align with WHO’s workforce and facility readiness priorities,^15^ which call for revising scopes of practice, implementing collaborative leadership training and ensuring procurement of essential equipment such as birthing stools and resources for upright births. Facility readiness assessments and workforce satisfaction surveys can serve as accountability tools.^18^

Conversely, facilitators identified in this study – such as strong interdisciplinary collaboration, clearly defined roles, effective referral pathways and mutual trust within healthcare teams – are critical enablers of continuity of care.^14^ These factors resonate with WHO’s guiding principles for integrated networks of care. Operationalising these facilitators through joint training, shared protocols and regular multidisciplinary reviews can strengthen implementation. Referral efficiency and staff-reported collaboration quality provide practical metrics for monitoring progress.

### Policy and global alignment

The convergence of structural, cultural and institutional barriers across diverse health system contexts underscores the need for coordinated multi-level strategies. The World Health Organization’s *Implementation Guide for Midwifery Models of Care* offers a roadmap through situation analysis, priority setting, operational planning and sustainable financing.^15^ Aligning national reforms with global frameworks such as the Sustainable Development Goals (SDG 3: Ensure healthy lives and promote well-being for all) is essential for accelerating progress. Embedding human rights and gender equality objectives within midwifery reforms, as advocated by the WHO,^14,15^ ensures that scale-up efforts advance equity and respect for women’s agencies. We encourage all policymakers to follow the WHO Implementation Guide for Midwifery Models of Care.^15^

### Strengths and limitations

A key strength of this study is the closeness to the text as described by the participants and the mirroring to the midwifery models of care.^12^ The use of FGD allowed for rich, in-depth data, enabling a nuanced understanding of both systemic and interpersonal factors influencing the integration of midwifery models of care in various health systems. Qualitative content analysis based on Graneheim and Lundman’s^24^ methodology ensured a rigorous and transparent analytic process, with attention to both manifest and latent content. The inclusion of diverse healthcare leaders’ perspectives with experiences working as midwives, nurses and medical doctors contributed to a more comprehensive view of interdisciplinary dynamics and contextual barriers and facilitators. However, the study also has limitations. The findings are based on a limited number of focus groups within specific geographical and institutional contexts, which may affect the transferability of the results to other settings.^25^ Some participants may have been reluctant to speak openly due to potential hierarchical dynamics within the group. Finally, the study focused primarily on healthcare leaders’ perspectives and did not include women’s or community members’ views, which are critical to fully understanding the acceptability and feasibility of midwifery models of care.

### Implications

Addressing barriers to midwifery-led care requires systemic transformation rather than incremental change. Key priorities include (1) infrastructure and resources: invest in health facility infrastructure and ensure equitable resource allocation to overcome geographic and structural limitations; (2) cultural engagement: implement culturally sensitive community outreach and male involvement initiatives to challenge restrictive norms and enhance women’s autonomy; (3) health literacy: launch targeted education campaigns and peer-led programmes to close knowledge gaps and increase demand for evidence-based practices; (4) policy integration: embed midwifery within national health strategies and establish accountability mechanisms to strengthen governance; (5) workforce empowerment: expand midwives’ decision-making authority and provide continuous professional development to improve care quality.

Further research should explore the implementation of dynamics at multiple levels, including gender relations, community engagement strategies and innovations for rural health systems.

## Conclusion

Transitioning to midwifery models of care requires structural change, not just training or task redistribution. Providers envisioned collaborative, team-based care grounded in respect and clear roles, yet institutional hierarchies, systemic constraints and misconceptions about midwives persist. Strengthening advocacy, integrating midwives into policy and fostering interdisciplinary collaboration are critical. Investments in leadership, staffing and professional development, alongside efforts to challenge entrenched attitudes, can create enabling environments. Evidence shows that when midwives are recognised and supported, maternal and neonatal outcomes improve significantly.
